# A patient-specific lumped-parameter model of coronary circulation

**DOI:** 10.1038/s41598-018-19164-w

**Published:** 2018-01-17

**Authors:** Zheng Duanmu, Min Yin, Xueling Fan, Xilan Yang, Xiaoyu Luo

**Affiliations:** 1 State Key Laboratory for Strength and Vibration of Mechanical Structures, School of Aerospace Engineering, Xi’an Jiaotong University, Xi’an, China; 2 The Second Affiliated Hospital of Nanjing Medical University, Nanjing, China; 3 School of Mathematics & Statistics, University of Glasgow, Glasgow, UK

## Abstract

A new lumped-parameter model for coronary hemodynamics is developed. This model is developed for the whole coronary network based on CT scans of a patient-specific geometry including the right coronary tree, which is absent in many previous mathematical models. The model adopts the structured tree model boundary conditions similar to the work of Olufsen *et al*., thus avoiding the necessity of invasive perfusion measurements. In addition, we also incorporated the effects of the head loss at the two inlets of the large coronary arteries for the first time. The head loss could explain the phenomenon of a sudden increase of the resistance at the inlet of coronary vessel. The estimated blood pressure and flow rate results from the model agree well with the clinical measurements. The computed impedances also match the experimental perfusion measurement. The effects of coronary arterial stenosis are considered and the fractional flow reserve and relative flow in the coronary vessels for a stenotic vessel computed in this model show good agreement with published experimental data. It is believed that the approach could be readily translated to clinical practice to facilitate real time clinical diagnosis.

## Introduction

Due to the increasing importance of understanding heart diseases and improving clinical treatments, modeling the human circulation has been a major research focus over the recent years. Frank^1^ was the first to have developed a resistance-compliance element windkessel, lumped parameter model for the circulation system. Following Frank’s model, the circulated network models were also promoted using a similar principle to electricity circuits. All these models can simulate the circulation using the left ventricular pressure as the inlet boundary condition. However, these models can only predict blood flows in the main systematic arteries. Moreover, these models need to involve many circuit elements to represent different physiological conditions^2–4^.

More advanced models incorporating smaller vessels have been developed as well. Wang *et al*.^5^ derived an improved network of the cardiovascular system by using an equivalent closed-loop analogue circuit model for the small segment of the coronary arteries. Mantero *et al*.^6^ investigated the cardiovascular hemodynamics under normal and pathological conditions in which the coronary bed was crudely modeled using lumped parameters. Pietrabissa *et al*.^7^ extended the lumped-parameter model of the coronary tree by adding a closed-loop model that includes the contribution of the body system. They were able to determine the terminal resistance using experimental perfusion measurements, and their coronary model was also used to assess coronary artery bypass graft surgery. The local wall shear stress and blood flow were observed before and after the bypass graft, in order to check whether the surgery was effective by using their virtual computation. However, the geometric morphology, such as the right coronary arteries, has been largely ignored. In addition, it is complicated and potentially harmful to perform perfusion measurements for patient-specific modelling.

A one-dimensional (1D) network system approach has also been widely studied^8,9^. Based on the wave transmission theory, Olufsen *et al*.^10–12^ developed a 1D model of the large systemic arteries using the structured tree impedances as the outflow boundary conditions. The space-time representation of the nonlinear 1D blood flow can be theoretically and numerically modelled by flow wave propagation in compliant vessels^13–15^. Using this approach, Olufsen and her co-workers^16,17^ studied blood flow in the pulmonary arteries. Further, they improved the mathematical and computational model of the pulmonary circulation by merging both arterial and venous trees. The terminal impedance of vessels as the boundary conditions was evaluated according to the radius of each root vessel. Qureshi *et al*.^18^ studied the pressure wave reflections in the pulmonary arteries using the conventional wave intensity analysis and a 1D computational model. It was found that the geometric properties of the pulmonary musculature play an important role in the system. Hence, patient-specific geometry should be included in the modelling of the coronary network.

Detailed arterial geometry and the fluid dynamics are best captured using the finite element (FE) approach and computational fluid dynamics (CFD). Liu *et al*.^19^ carried out an 3D FE simulation of the main circle of Willis combined with a lumped parameter model boundary condition, and the result showed good agreement with the Doppler ultrasound measurements. Using a FE approach, Al-Hassan *et al*.^20^ and Kim *et al*.^21^ studied the three-dimensional whole coronary system using more detailed geometries reconstructed from computed tomography (CT). Each coronary outlet boundary condition was coupled to a lumped-parameter coronary bed model and the inlet was assigned to the systemic circulation. However, such a 3D approach requires a complex model reconstruction and the geometry smoothing from CT scans can be extremely time consuming.

Stenosis (a reduced cross-sectional area) in the circulation system can result in many arterial diseases. Zhang *et al*.^22^ studied the human common stenotic carotid arteries bifurcation. Sui *et al*.^23^ used clinical measurement and CFD study to estimate the trend of hemodynamic parameters near atherosclerotic plaques stenosis in the carotid arteries. A potential threshold value of the CFD-based pressure gradient could serve as an additional factor for improving the accuracy of grading the severity of the stenosis^24^. The stenosis of a 0D coronary model was presented by changing the parameter to mimic the reduction of cross-section area^5^. The Fractional Flow Reserve (FFR), defined as the ratio of pressures distal and proximal to the lesion, is an important indicator in clinical diagnosis of stenosis^25^.

In this paper, we aim to develop an efficient lumped-parameter model. Our model includes the whole coronary network based on CT scans of a patient-specific geometry including the right coronary tree, which is absent in many previous mathematical models^5,7^. The model adopts the structured tree model boundary conditions based on the work of Olufsen *et al*.^11^, thus avoiding invasive perfusion measurements. We also incorporate the effects of the head loss at the two inlets of the large coronary arteries. In addition, effects of stenosis of different degrees in coronary vessels are modelled and analyzed using FFR.

## Results

### The pressure and the flow rate in coronaries

**Figure 1:**
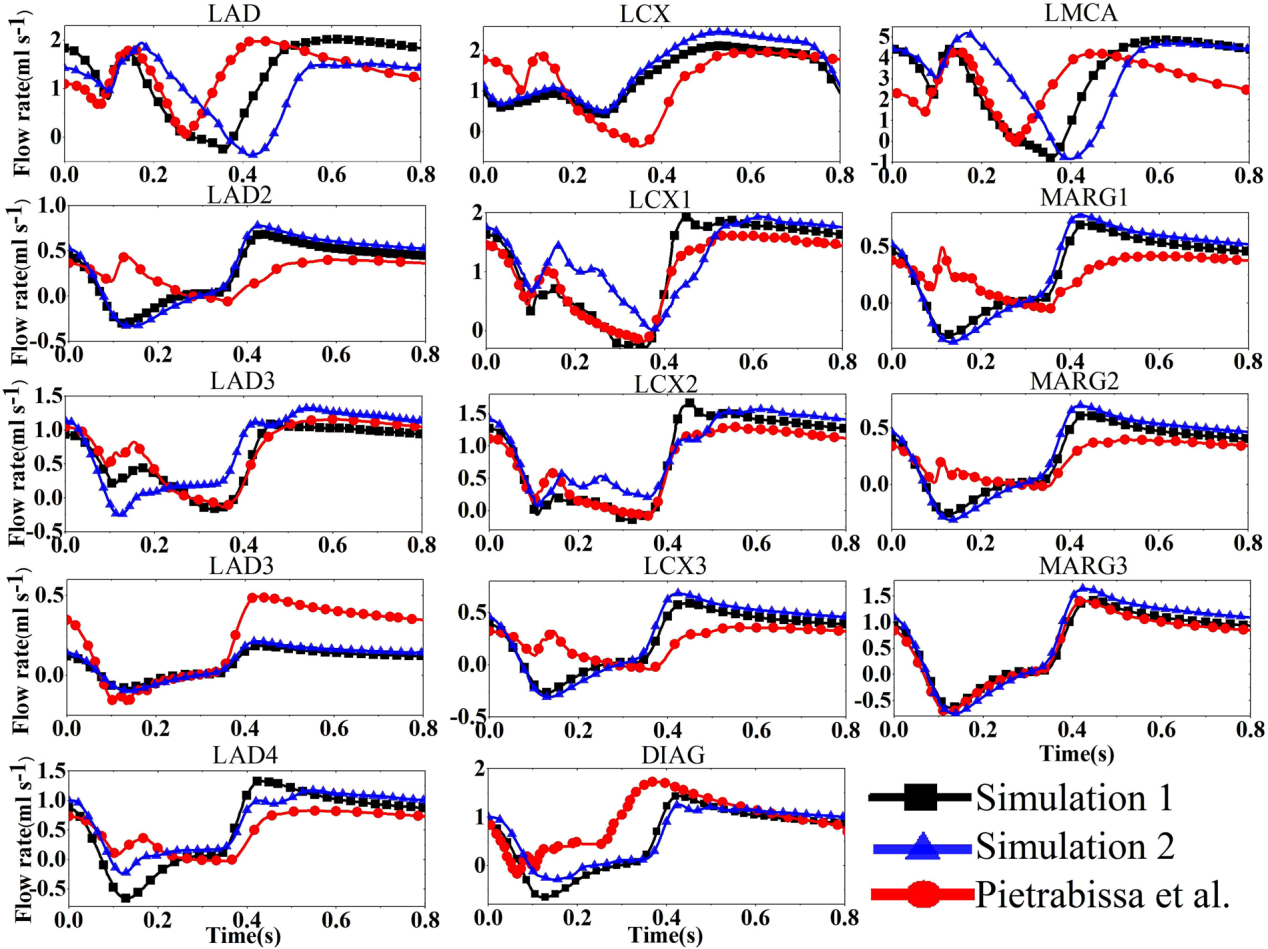
Simulated flow rates over one heart beat along different sections of the left coronary arteries, based on our geometry (Simulation 1), on the geometry by Pietrabissa *et al*.^7^ (Simulation 2), and the model result by Pietrabissa *et al*. based on a constant resistance.

We first run a simulation based on our geometry derived from the CT scans (we refer to this as Simulation 1). The flow rates along different sections of the left coronary arteries are plotted against time in Fig. 1. The results are compared with the previous result by Pietrabissa *et al*.^7^. To separate the model differences and the patient-specific geometry difference, we also use their geometric parameters in our model and the results are also shown in Fig. 1 (Simulation 2). Note that Pietrabissa *et al*.^7^ added an arbitrary resistance in their model, while our resistance is based on a rational estimation of the flow-dependent head loss. It is interesting to see that the difference in patient geometries only affects the flow rate in larger arteries (i.e., LAD, LCX, and LMCA). However, the model difference (using different resistance) is significant in most of the arteries. The diastolic dominated flow in the left coronary tree shows a typical decline during early systole. The wave forms changes slightly with locations. Due to the myocardial contraction, the blood flow rate drops at the beginning of systole and increases during diastole. We observe that the variation of coronary flow is location dependent due to the vascular pressure change.

**Figure 2:**
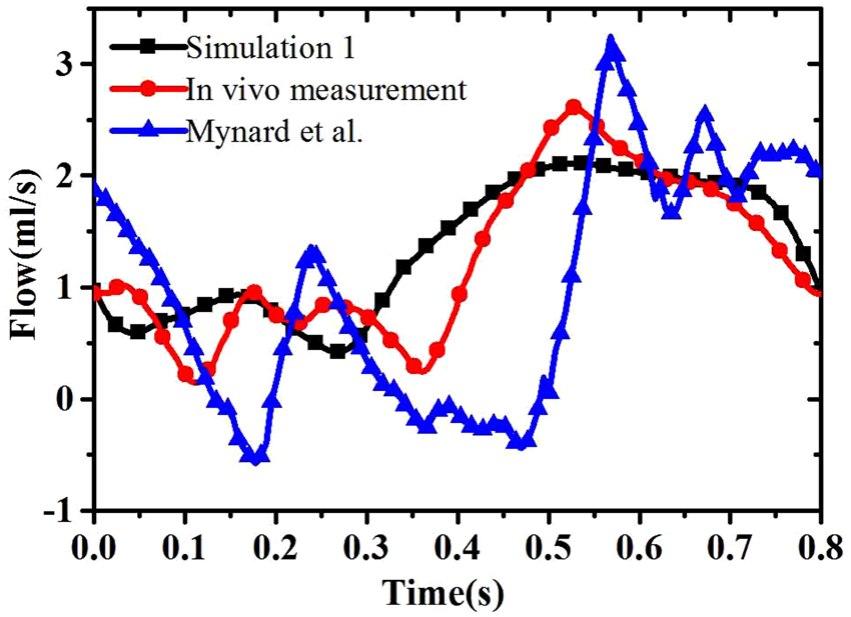
Comparison of the flow along LCX from Simulation 1, *in vivo* measurement of the same patient, and *in vivo* measurement of a different patient by Mynard *et al*.^26^.

The computed flow waveform in LCX is also compared directly to our own *in vivo* measurement using a Doppler catheter (with the patient’s permission), as well as with the published *in vivo* measurements by Mynard *et al*.^26^, shown in Fig. 2. There is an overall agreement between our simulations and the *in vivo* data. Compared to the *in vivo* data, the simulated coronary hemodynamics is less wavy, presumably due to the fact that we ignored wave reflections in the system. Figure 2 also shows that patient-specific modelling is important in LCX.

### Effect of head losses

**Figure 3:**
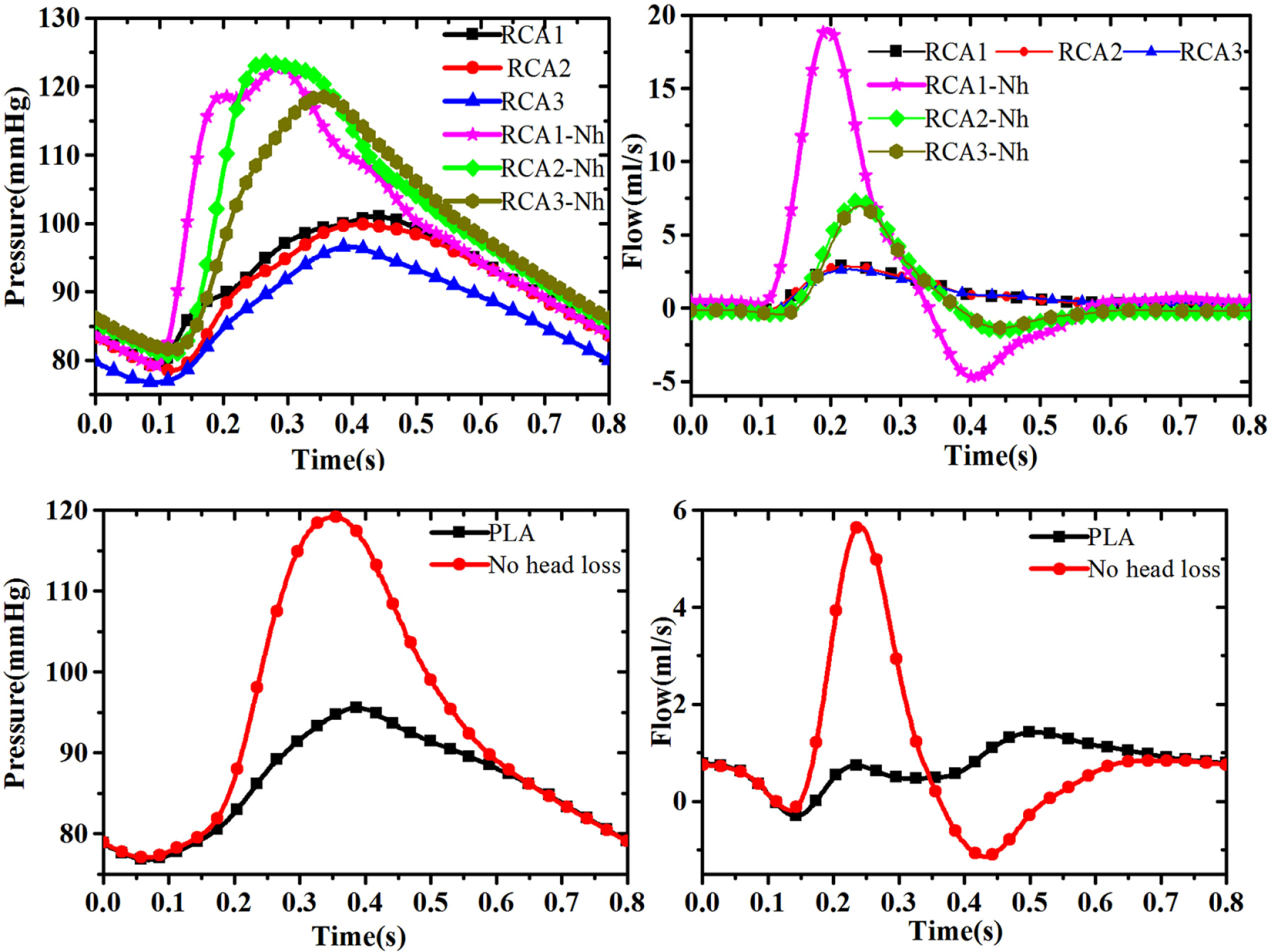
The pressure and flow rate waveforms, with or without head loss, along the RCA segments (top), where Nh in RCA stands for no head loss, and along the PLA (bottom).

We now study the effect of the head loss. The pressure and flow in both the RCA and PLA (with and without the local head losses) are shown in Fig. 3. We can see that the difference between the right and left coronary flow demonstrates the influence of the intra-myocardial pressure as a feedback to the cardiac system. We remark that the flow waveforms in PLA and RCA are much smaller than those without the head loss. Figure 3 shows the pressure and the corresponding flow waveforms in these vessels. We note that the pressure with head loss is in the physiological range. The phenomenon of the sudden increase of the resistance at the inlets of LAD and RCA was observed previously but not explained^5,7^. Our model suggests that this sharp increase of the resistance is due to the head losses.

### Effect of coronary stenosis and FFR

**Figure 4:**
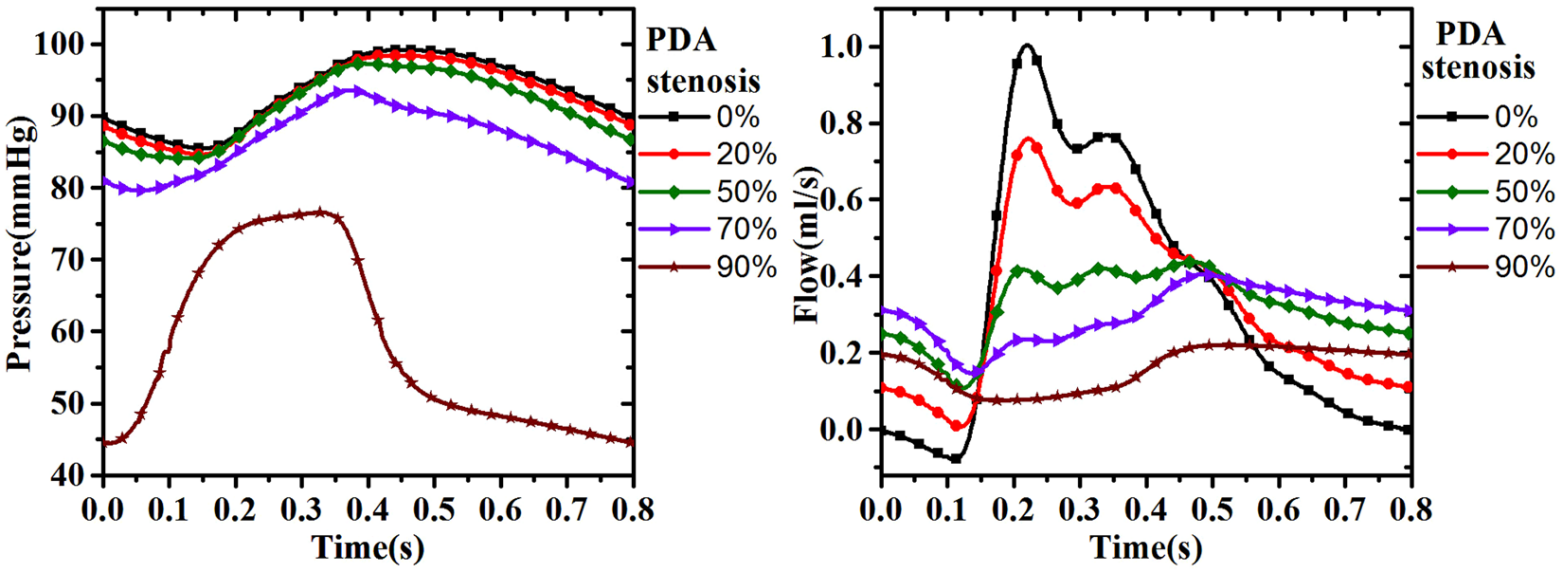
The pressure (left) and flow (right) for different degrees of stenosis in PDA.

The effect of a stenosis is investigated in the coronary system using our model. We define the stenosis degree *α* using a cross-sectional area reduction: *α* = *A*_*S*_/*A*_0_, where *A*_0_ is the normal cross-sectional area and *A*_*S*_ is the stenotic one. Then the resistance, capacitance, and the inductance of a stenotic vessel become^5^,

1 $${R}_{s}={R}_{0}{\alpha }^{-2}\quad {C}_{s}={C}_{0}{\alpha }^{\mathrm{3/2}}\quad {L}_{s}={L}_{0}{\alpha }^{-1},$$

where *R*_0_, *C*_0_, *L*_0_ denote the resistance, capacitance, and the inductance of the non-stenotic vessel, respectively. Here we assume that *α* = 100 − *p*, where *p* is the percentage of the area reduction. Figure 4 shows the details of the flow and pressure in the right coronary PDA, with the percentage of stenosis changing from 20% to 90%. The waveform of the flow in PDA decreases with the percentage of the stenosis in early systole. However, in diastole there is no significant flow drop for a stenosis below 50%. In cases of 70% or larger stenosis, the flow is greatly reduced in the late systole cardiac cycle. The results suggest that the pressure is perhaps a better indicator of the stenosis compared with the flow rate. This is because the pressure in PDA reduces monotonically with the narrowing rate, but the flow rate changes more erratically.

**Figure 5:**
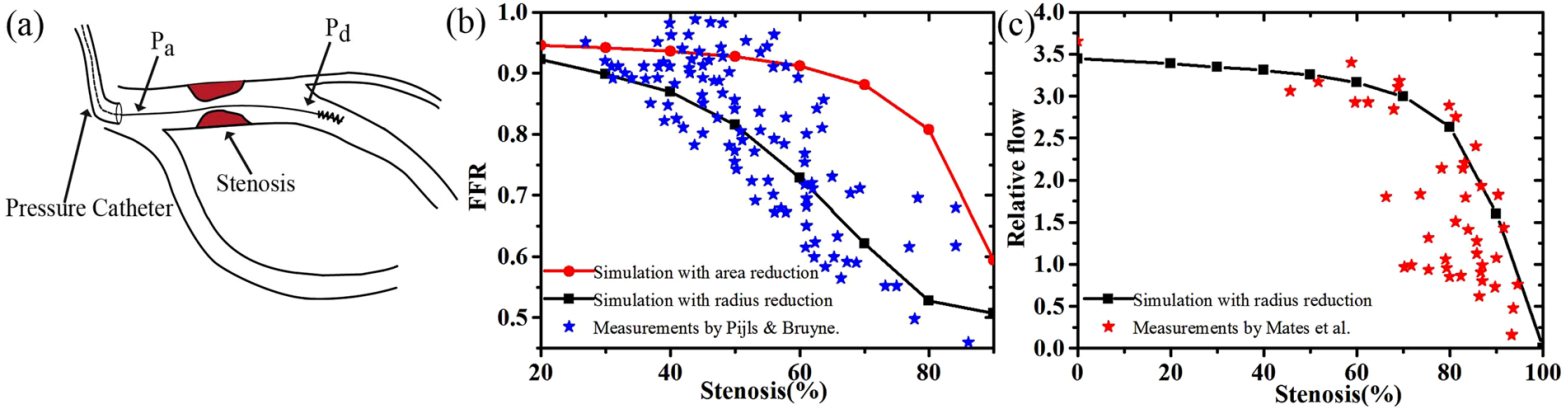
(**a**) A typical display of coronary stenosis and FFR. (**b**) PDA of our simulations versus stenoses, compared with the measurements by Pijls & Bruyne^27^, (**c**)non-dimensional flow simulation versus stenosis, compared with the measurements by Mates *et al*.^28^.

FFR can guide the percutaneous coronary intervention (PCI) with drug-eluting stents in patients with coronary artery stenosis. As can be seen in Fig. 5(a), FFR can be expressed as the ratio of the pressure distal to the lesion (*p*_*d*_) and the pressure (*p*_*a*_) proximal to the lesion^25^:

2 $$\,{\rm{FFR}}\,=\frac{{p}_{d}}{{p}_{a}}\mathrm{.}$$

When FFR is smaller than 0.8 and the stenosis is less than 50%, stenting is recommended^20^. FFR at different levels of stenosis in PDA is shown in Fig. 5(b). The relation of FFR and stenosis was studied by Pijls & Bruyne^27^ with clinical statistic data obtained from 221 patients before angioplasty or during diagnostic coronary angiography.

Figure 5(b) shows that the present area reduction model gives a general agreement with the clinical results. Note that the clinical definition of stenosis is based on the radius ratio (i.e., *α* = *r*_*s*_/*r*, where *r* and *r*_*s*_ are the normal and stenotic radii) instead of the area ratio. Therefore, the simulation result with the radius reduction is also shown for comparison. Our model can also predict the relative flow against degrees of stenosis, shown in Fig. 5(c). Again, the result agrees well with the experimental measurements by Mates *et al*.^28^.

## Discussion

In this study, we propose a new model of simulating the coronary hemodynamics. Three new features are included in this model. Firstly, the whole coronary model geometry is reconstructed from CT scans of a real patient. Secondly, our work demonstrated that head loss at the inlets of the coronary vessels, where sudden diameter change occurs, can significantly improve the accuracy of the results. Finally, the model parameters of the root impedance are obtained non-invasively based on a structured tree model. Using this model, the coronary blood flow rate and pressure of each vessel can be assessed quickly without time-consuming computations, and the results agree well with clinical observations and experiment data. In addition, effects of stenosis on the coronary flow are considered.

Our model yields reasonable results by considering the head loss. The FFR and relative flow in the coronary vessels for a stenotic vessel computed in this model also show good agreement with the experimental data published previously, although it is noted that the experiment data has a high level of noise.

Finally, limitations of the model are: Firstly, the model does not include specific dynamic input of the heart function. Additionally, we assumed that the vessel segments are rigid tubes, thus the model cannot predict wave propagations in the system. We also mention the limitation that measurements of coronary flow were not performed in some specific situation including an artery with large side branch. More sophisticated models can be developed to remove these limitations, although the challenges are to obtain patient-specific material properties and the spatial deformation of the whole heart.

## Methods

### Patient-specific coronary tree

The geometries of the coronary tree were obtained using the 64 slices CT scans of an adult male aged 65 with hypertension in the Second Affiliated Hospital of Nanjing Medical University, with the permission of the patient. We divide the coronary tree into several segments according to the American Heart Association standard^29^ and the clinical data^30^.

**Figure 6:**
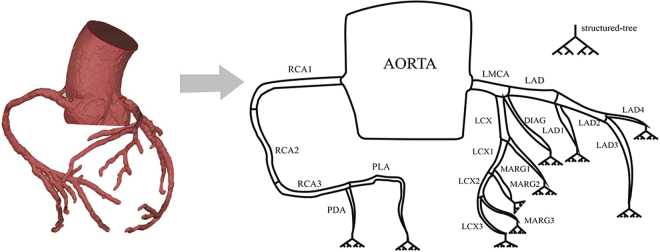
Reconstruction of the model, based on the CT scan (left) to the model representation of the whole coronary tree (right). The model is clinically divided into segments with names according to the CT terminology. The terminal branches are connected to a structured tree model representing the capillary bed in the myocardium.

As shown in Fig. 6, all the vessel terminals are connected to a structured tree model. The left and right coronary trees are linked to the aorta and the head loss resistance (*R*_*h*_) at the junctions is included. The long right coronary artery (RCA) is further divided into three segments as in clinical practice. In this model, we consider both the left coronary artery (LCA) and the right coronary artery (RCA). This is because in addition to supplying the right ventricle with blood, RCA also supplies 25% to 35% of the blood flow to the left ventricle (LV)^31^. In particular, for right dominant patients, the RCA plays a key role in heart diseases.

**Figure 7:**
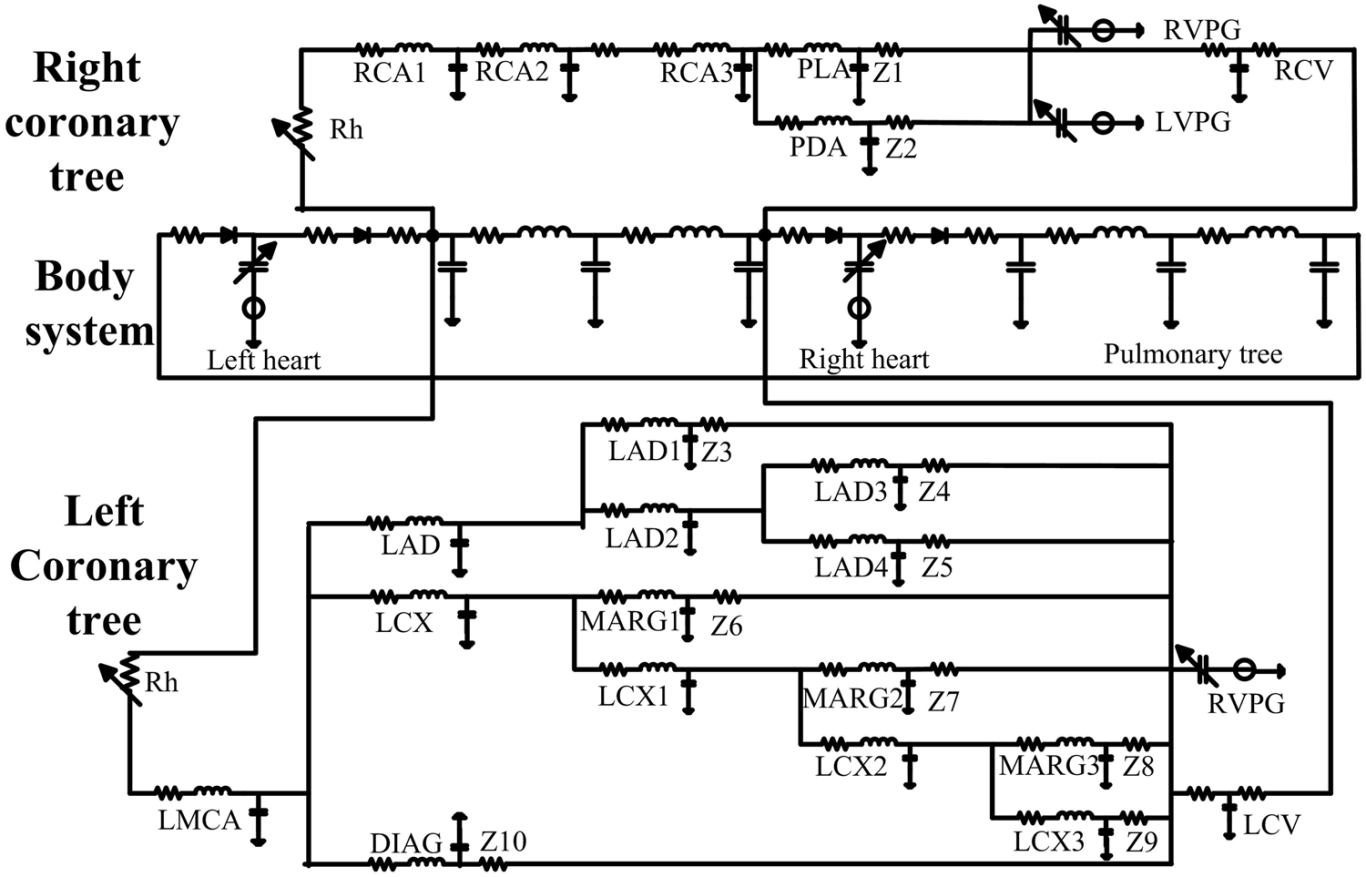
The coronary tree is represented by a closed loop cardiovascular system, where *R*_*h*_ is the variable resistance due to head loss, and Z1 to Z10 are the root impedances for the structured tree models attached at the end of the coronary vessels.

The analogous circuit network for the whole coronary tree is illustrated in Fig. 7. In the network, all the terminals of the coronary arteries are assigned to a root impedance computed from the structured tree model. Similar to the study by Pietrabissa *et al*.^7^, the network contains a closed-loop model of the cardiovascular system. The intra-myocardial pressures (at RVPG and LVPG) act as the pressure generators, which are chosen to be third or half of the ventricular pressure depending on the coronary vessel locations in the heart^26^. The coronary veins are simulated in the model and linked to the right atrium simultaneously. The systemic and pulmonary circulations are also included. The right ventricular pressure generator (RVPG) and the left ventricular pressure generator (LVPG) are feedback terms. The left coronary veins (LCV) and the right coronary veins (RCV) are also connected to the systems as the terminal impedances (Z).

### Mathematics of the lumped-parameter model

We assume the blood to be Newtonian fluids and the vessels to be cylindrical tubes^32^. The governing equations of the flow in each vessel are^33^

3 $$-{\rm{\Delta }}p=L\frac{\partial q}{\partial t}+Rq,$$

4 $$q=C\frac{\partial p}{\partial t},$$

where *p* is the pressure, Δ*p* is the pressure drop, and *q* is the flow rate. The other parameters are: hydraulic resistance *R*, the elastic capacitance of each vessel *C* and the inertia of the blood flow *L*. Thus, analogous to resistance, capacitance, inductance in a circuit, we have^5,7^

5 $$R=\frac{128\mu l}{\pi {D}^{4}},\quad C=\frac{\pi {D}^{3}l}{4Gh},\quad L=\frac{4\rho l}{\pi {D}^{2}},$$

where *μ* is the blood viscosity, *ρ* is the density, *G* is the Young’s modulus of the vessels, *l* is the segment length, *D* is the diameter, and *h* is the wall thickness of the vessel. The parameters of all the vessels estimated from the CT data are listed in Table 1, where the heart rate of 75 beats per minute (cardiac cycle of 0.8 s) was measured. We also chose *μ* = 4 × 10^−3^ *kg* *·* *m*^−1^*s* ^−1^, *ρ* = 1.04 × 10^3^ *kg*/*m*^3^ and *G* = 2 × 10^5^ Pa. The wall thickness *h* is 0.08*D*, with *D* being the vessel diameter.

**Table 1:** Parameters of the patient-specific coronary model estimated from the CT data.

Branch	Resistance(R) (mmHg·s·ml^−1^)	Inductance(L) (mmHg·ml^−1^)	Capacitance(C) (ml·mmHg^−1^)
RCA	1.64	0.12	0.0064
PDA	2.31	0.079	0.011
PLA	1.31	0.064	0.017
LMCA	0.14	0.018	0.0035
LAD	0.5	0.144	0.0017
LAD1	0.24	0.023	0.0023
LAD2	0.87	0.067	0.0045
LAD3	0.43	0.031	0.00018
LAD4	0.63	0.041	0.0019
DIAG	3.19	0.12	0.0013
LCX	0.17	0.16	0.0017
LCX1	0.14	0.013	0.0013
LCX2	0.19	0.017	0.00015
LCX3	0.38	0.033	0.0027
MARG1	0.71	0.049	0.0027
MARG2	1.17	0.064	0.0021
MARG3	1.94	0.081	0.0016

As the blood supplies are from heart ventricles, the left ventricle (LV) and right ventricular (RV) pumping functions are chosen, following^34,35^ to be

6 $${P}_{v}=\{\begin{array}{ll}{U}_{0}\sigma (t)+E(V-{V}_{0})+{R}_{v}\dot{V} & {\rm{systole}}\\ {E}_{d}(V-{V}_{0}) & {\rm{diastole}}\end{array}$$

where *P*_*v*_ is the ventricular pressure, *U*_0_ is a peak isovolumetric pressure at volume *V*_0_, *E* is the time-varying elastance, the activation function *σ*(*t*) = [1 − *cos*(2*πt*/*t*_*s*_)], *t*_*s*_ is the systolic period, *V* is the reference volume, $\dot{V}$ is the rate of volume change, *R*_*v*_ is the resistance of ventricular myocardium, and *E*_*d*_ is the passive elastance. All the pumping function model parameters are listed in Table 2.

**Table 2:** The parameters for the ventricular pressure modelling.

	Left ventricle	Right ventricle
*R*_*v*_ (mmHg·s·ml^−1^)	0.0800	0.0175
*U* (mmHg)	50	24
*E*_*d*_ (mmHg·s·ml^−1^)	0.10	0.03
*V*_0_ (ml)	11.29	3.33

### The terminal impedance

The terminal resistance of each vessel is linked to the capillaries downstream. In many previous models, the terminal resistance is estimated using a three-element Windkessel model^36^, where the total resistance and the compliance of the vascular bed need to be measured, which is often difficult to do. Hence, in this paper, we seek an alternative approach.

To estimate the terminal resistance, compliance and the inertia of the whole coronary tree, we made use of the structured tree model by Olufsen *et al*.^11^, as well as the perfusion measurements^7^. For simplicity, we only consider steady flow and the bifurcations of a symmetric structured tree when the diameter of vessels is less than 0.01 mm. These small arteries are treated as a structured network of straight vessels. The impedance at a vessel bifurcation is:

7 $$\frac{1}{{Z}_{p}}=\frac{1}{{Z}_{d1}}+\frac{1}{{Z}_{d2}}\mathrm{.}$$

(7) can be solved recursively to obtain the root impedance of the terminal vessel of the vascular beds^11^.

Hence, the terminal impedance for a symmetric tree of N generations is

8 $$Z=\frac{8\mu \lambda }{\pi {r}_{0}^{3}}\frac{2{\alpha }^{3}-{(\frac{1}{2\alpha })}^{N}}{2{\alpha }^{3}-1},$$

where *λ* = 50 is a constant defining the length to radius ratio in small arteries^10,37^, *r*_0_ is the original root radius, and *α* = *r*_*d*_/*r*_*p*_ = 0.778 is the ratio of the daughter vessel radius (*r*_*d*_) and the parent vessel radius (*r*_*p*_) for a symmetric tree network when the arterial taper exponent rate is chosen to be 2.76^11,38,39^.

The values of *Z* at different ends of the coronary vessels (Z1-Z10) are listed in Table 3. These are compared with the corresponding perfusion resistance measurements^5,7^. A similar trend is seen between our estimations and the measurements.

**Table 3:** Comparison of the terminal impedance estimated from the structured tree model (8) and published perfusion data. No published data are found for Z1 and Z2.

Vessel name	Structured tree model *r*_0_(mm), Z(mmHg·s/ml)	Perfusion data^5^ (mmHg·s/ml)
PLA	*r*_0_ = 1.25, Z1 = 46.9	
PDA	*r*_0_ = 1.26, Z2 = 53.8	
LAD1	*r*_0_ = 0.89, Z3 = 108.7	102.7
LAD3	*r*_0_ = 0.71, Z4 = 289.8	291.1
LAD4	*r*_0_ = 0.77, Z5 = 251.9	204.3
MARG1	*r*_0_ = 1.08, Z6 = 67.4	53.8
MARG2	*r*_0_ = 0.86, Z7 = 116.7	114.1
MARG3	*r*_0_ = 0.79, Z8 = 226.3	202.0
LCX3	*r*_0_ = 0.77, Z9 = 251.7	230.9
DIAG	*r*_0_ = 1.07, Z10 = 70.6	49.1

### Evaluation of the head loss

**Figure 8:**
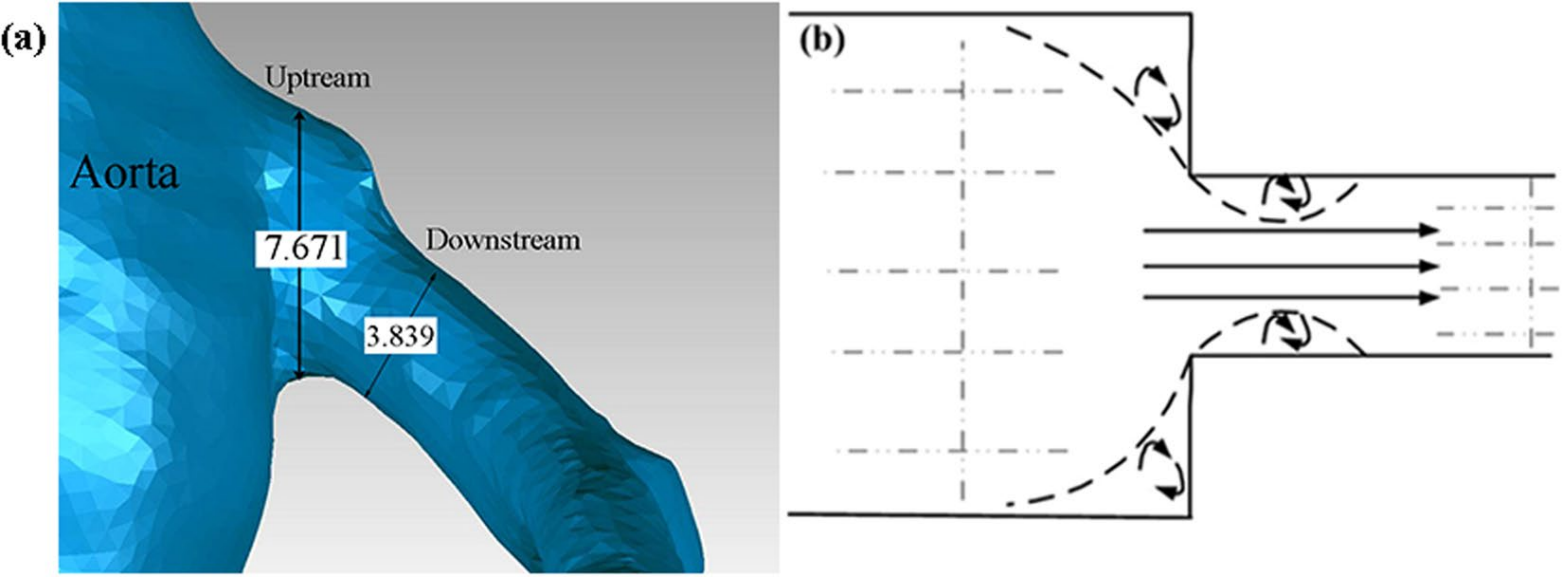
Locations of head loss in both the right and left coronary trees and the head loss due to a cross-sectional area constriction.

Energy losses associated with vessel structures and the loss coefficient can be incorporated into Bernoulli’s equations^9^. To model the head loss (a sudden pressure-drop due to cross-sectional area change) at the inlets of the coronary arteries, as shown in Fig. 8, we estimate the pressure drop from

9 $${\rm{\Delta }}p=\frac{\xi \rho {v}^{2}}{2},$$

where *ξ* is the local resistance, estimated to be 2.88 from the empirical formula *ξ* = 288/Re by Fester *et al*.^40^, for a vessel constriction diameter ratio of 0.5 and Re = 100, and *v* is the mean velocity. The resistance *R*_*h*_ due to the head loss is then

10 $${R}_{h}=\frac{{\rm{\Delta }}p}{q}=\frac{\xi \rho }{2{A}^{2}}q\mathrm{.}$$

(10) shows that the local head loss in the coronary circulation system is equivalent to a variable resistance that is proportional to the flow rate *q*.

### The geometry measurements and flow experiments

The right and left coronary trees are reconstructed from the *in vivo* data from a male patient (age 61, 80 kgs). The GE 16-slice CT angiography was performed and the medical image software (GE,AW) was used for visualization and measuring vessel length and radius. The blood flow experimental data of left circumflex branch coronary vessels was measured by an intra-coronary Doppler guide wire (FloWire, 1400-Floppy). All the experiments were performed by The Second Affiliated Hospital of Nanjing Medical University, China. All the procedures were conducted according to guidelines established by The Second Affiliated Hospital of Nanjing medical University, and every effort was made to minimize patient suffering. This study was approved by the Human Experiment Committee of The Second Affiliated Hospital of Nanjing Medical University. The patient provided written consent and it was informed consent that was obtained in this paper.

## Conclusion

In this work, a new lumped-parameter model based on the CT scans of coronary circulation is proposed. This model incorporates the effect of head loss at the junctions of inlets for the first time. Furthermore, the terminal resistances of the coronary arteries are computed by using structured tree models without invasive perfusion measurements. The results generated by our model are in good agreement with the previous studies and experimental data. In addition, the result shows that the estimated pressure and flow of different stenosis provide a useful non-invasive method for cardiac disease diagnosis. Our model can be used to provide detailed flow information of a patient-specific whole coronary circulation system due to various diseases, such as stenosis and local stiffness change, and thus can be readily translated into clinical practice and facilitate real-time clinical diagnoses.
